# Histochemical Detection of Collagen Fibers by Sirius Red/Fast Green Is More Sensitive than van Gieson or Sirius Red Alone in Normal and Inflamed Rat Colon

**DOI:** 10.1371/journal.pone.0144630

**Published:** 2015-12-16

**Authors:** Cristina Segnani, Chiara Ippolito, Luca Antonioli, Carolina Pellegrini, Corrado Blandizzi, Amelio Dolfi, Nunzia Bernardini

**Affiliations:** 1 Unit of Histology and Medical Embryology, Department of Clinical and Experimental Medicine, University of Pisa, Pisa, Italy; 2 Division of Pharmacology, Department of Clinical and Experimental Medicine, University of Pisa, Pisa, Italy; Scuola Superiore Sant'Anna, ITALY

## Abstract

Collagen detection in histological sections and its quantitative estimation by computer-aided image analysis represent important procedures to assess tissue localization and distribution of connective fibers. Different histochemical approaches have been proposed to detect and quantify collagen deposition in paraffin slices with different degrees of satisfaction. The present study was performed to compare the qualitative and quantitative efficiency of three histochemical methods available for collagen staining in paraffin sections of colon. van Gieson, Sirius Red and Sirius Red/Fast Green stainings were carried out for collagen detection and quantitative estimation by morphometric image analysis in colonic specimens from normal rats or animals with 2,4-dinitrobenzenesulfonic acid (DNBS) induced colitis. Haematoxylin/eosin staining was carried out to assess tissue morphology and histopathological lesions. Among the three investigated methods, Sirius Red/Fast Green staining allowed to best highlight well-defined red-stained collagen fibers and to obtain the highest quantitative results by morphometric image analysis in both normal and inflamed colon. Collagen fibers, which stood out against the green-stained non-collagen components, could be clearly appreciated, even in their thinner networks, within all layers of normal or inflamed colonic wall. The present study provides evidence that, as compared with Sirius Red alone or van Gieson staining, the Sirius Red/Fast Green method is the most sensitive, in terms of both qualitative and quantitative evaluation of collagen fibers, in paraffin sections of both normal and inflamed colon.

## Introduction

Collagen is one of the major component in the adult gastro-intestinal wall, starting from the early stages of organogenesis [1] up to normal adult life and pathological conditions, such as inflammatory-induced bowel fibrosis [2–4]. Distribution and quantitative estimation of collagen fibers can be assessed by several morphological methods applied on tissue sections. Among these, histochemistry represents a simply and quick procedure for detecting total collagen tissue content. In this regard, picro-sirius red dyes are widely used due to their specific reactivity to most collagen types [5–7] and, therefore, they have been largely employed for quantitative estimations of tissue fibrosis [6–8] in several organs, such as liver [9–11], lung [12–13], kidney [14–16] and gastrointestinal tract [17–18]. In addition, since 1985 Sirius Red has been combined with Fast Green for histochemical staining and quantification of collagens and total proteins, respectively, in paraffin tissue sections, by biochemical/spectrophotometric [19–20] or microscopic image analysis [21–23].

The purpose of the present investigation was to compare three conventional histochemical methods, suitable for collagen staining, in terms of their efficiency for qualitative and quantitative detection of collagen fibers by computer-aided morphometric image analysis in light microscopy. In particular, staining protocols, carried out by van Gieson and Sirius Red, alone or combined with Fast Green, were compared for their capability of revealing and allowing the quantification of collagen deposition in paraffin sections of colon from normal rats or with colitis induced by 2,4-dinitrobenzenesulfonic acid (DNBS).

## Materials and Methods

### Animals

Albino male Sprague—Dawley rats, 200–250 g body weight, were used throughout the study. The animals were fed standard laboratory chow and tap water ad libitum, and were not employed for at least one week after their delivery to the laboratory. They were housed, three in a cage, in temperature-controlled rooms on a 12-h light cycle at 22–24°C and 50–60% humidity. All experimental protocols were approved by the Animal Care and Use Committee of the University of Pisa, and were in compliance with the national and European guidelines for handling and use of experimental animals. All efforts were made to minimize stress and suffering. Colitis was induced in accordance with the method previously described by Fornai et al. [24]. Briefly, animals (n = 6) were anesthetized with isoflurane and 30 mg of DNBS in 0.25 ml of 50% ethanol were administered intrarectally via a polyethylene PE-60 catheter inserted 8 cm proximal to the anus. Normal rats (n = 6) were treated in a similar manner with 0.25 ml of saline. Animals were evaluated on day 6 from DNBS administration to assess colonic inflammation and fibrosis. At this time, the animals were euthanized by overdose of isoflurane, and the colon was excised and processed for macroscopic damage score as well as for histological analysis. The evaluation of colonic inflammation severity was performed both macroscopically and histologically, in accordance with the criteria previously reported in our laboratories by Antonioli et al. [25] and Ippolito et al [23]. Microscopic evaluations were carried out by light microscopy on haematoxylin- and eosin-stained sections obtained from whole-wall specimens, taken from normal colon or from a region of inflamed colon immediately adjacent to the gross macroscopic damage.

### Histological analysis

Colonic samples were immediately fixed in cold 4% neutral formalin diluted in phosphate-buffered saline at 4°C and routinely processed for paraffin embedding and cross-sectioned to obtain 3 μm-thick sections with circular layer and myenteric ganglia cut longitudinally. Before use, sections were deparaffinized, rehydrated and processed for routine haematoxylin/eosin, and histochemical staining.

Tissue collagen deposition was detected by applying the following histochemical staining protocols:

### van Gieson

Colonic sections were treated with haematoxylin for 10 min, washed in tap water and incubated for 2 min in a picrofuchsia acid solution (1% acid fuchsin in acqueous saturated picric acid). Sections, dehydrated and mounted with DPX Mounting, showed collagen fibers pink-stained, nuclei and muscle black- and yellow-coloured, respectively.

### Sirius Red

The slides were incubated with a 0.1% Sirius Red solution dissolved in acqueous saturated picric acid for 1 hour, washed in acidified water (0.5% hydrogen chloride), dehydrated and mounted with DPX Mounting. Collagen and non-collagen components were red- and orange-stained, respectively.

### Sirius Red/Fast Green

Collagen fibers were detected in colonic tissues as previously reported [23]. Briefly, colonic samples were incubated in 0.04% Fast Green for 15 min, washed with distilled water and then incubated in 0.1% Fast Green and 0.04% Sirius Red in saturated picric acid for 30 min. Then, they were dehydrated and mounted with DPX Mounting. Collagen fibers appeared red, while the non-collagen proteins were green.

Quantitative estimations of histochemical stainings were carried out independently by two blind investigators (C.S. and C.I.). Each investigator analyzed all tissue specimens under study. The respective values were then averaged and plotted in graphs in accordance with previously described criteria [23]. Briefly, for each animal, 5 randomly selected microscopic fields from 3 non-adjacent sections, which were selected every 18 sections in order to ensure the evaluation of colonic samples with an average thickness of 160 μm, were captured by a Leica DMRB microscope equipped with the digital camera DFC480. All images, which were captured with 100x or 400x objective for studying the whole wall or *tunica muscularis*, respectively, were quantitatively estimated for collagen fibers and cellular non-collagen proteins in double Sirius Red/Fast Green staining, within the respective colonic areas. To detect the specific threshold of different colors (e.g., pink/red for collagen fibers and green for non-collagen proteins), a square was applied upon the color of interest and recorded by Image Analysis System ‘L.A.S. software v.4’. Positive tissue areas were automatically estimated on the basis of the total pixel number and intensity. The whole wall and *tunica muscularis* areas were manually circumscribed and automatically calculated. Data were expressed as percentage of Σ of positive-stained area / Σ of tissue area examined of whole wall or *tunica muscularis* in three colonic sections (5 fields/each) for each animal.

### Statistical analysis

Comparisons between groups were performed using the Wilcoxon signed rank test for paired data. All data are given in scatter plot graphs as mean ± SEM (n = 6 animals/group) and a P-value ≤ 0.05 was considered statistically significant. All statistical analysis were carried out using the Statistical Package for Social Science (IBM Corp. Released 2013. IBM SPSS Statistics for Windows, Version 22.0. Armonk, NY).

## Results

### Evaluation of collagen fibers in normal colon

**Fig 1:**
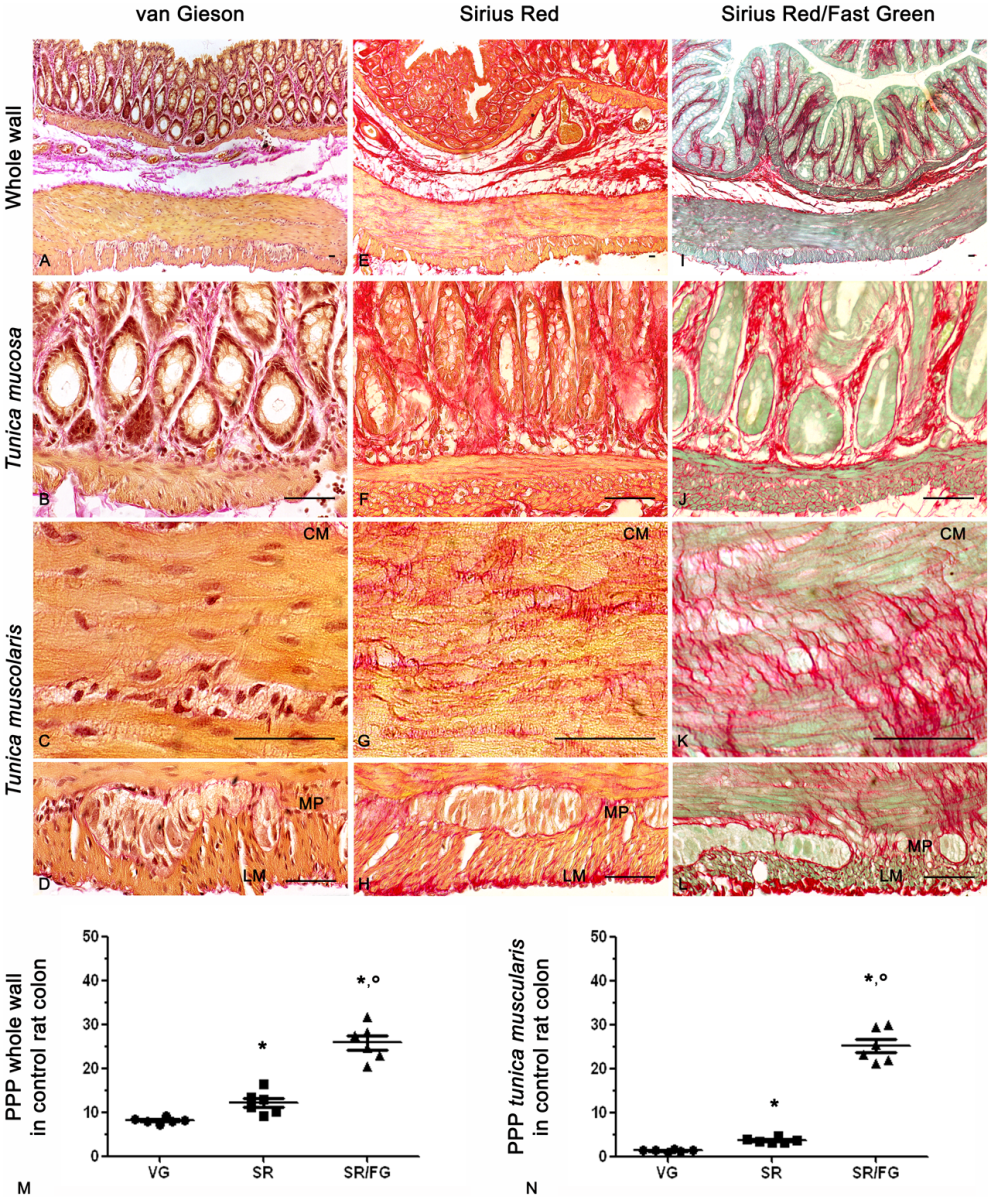
Representative photomicrographs of full-thickness normal rat colon showing the distribution pattern of collagen fibers stained with van Gieson (VG, A-D), Sirius Red (SR, E-H) and Sirius Red/Fast Green (SR/FG, I-L). CM and LM: circular and longitudinal muscle, respectively; MP: myenteric plexus. Scale bars represent 50 μm. Scatter plots show the percentage of positive pixel (PPP) of collagen ± SEM obtained from 6 rats in the whole colonic wall (M) and *tunica muscularis* (N). *,° P = 0.028 *versus* VG and SR, respectively.

Normal colon displayed appreciable amounts of van Gieson pink-stained collagen fibers in the *tunica mucosa* and *submucosa* (Fig 1A and 1B). By contrast, great amounts of tightly packed collagen fibers were highlighted by Sirius Red staining at the level of *tunica mucosa* and *submucosa* (Fig 1E and 1F) as well as in the circular and longitudinal layers of *tunica muscularis* and along the myenteric ridge encasing most of the ganglia (Fig 1G and 1H). Sirius Red/Fast Green staining allowed to clearly visualize collagen fibers, which appeared as well-defined, red-stained fibrillary elements, standing out from green-stained non-collagen components. Sirius Red/Fast Green-stained sections showed consistent amounts of collagen fibers, which appeared compacted in bundles of different thickness at level of the *tunica mucosa* and *submucosa* (Fig 1I and 1J), or as fine networks among smooth muscle cells of the *muscularis mucosae* and *tunica muscularis* (Fig 1I, 1K and 1L). Sirius Red/Fast Green allowed to detect higher amounts of collagen fibers as compared with van Gieson and Sirius Red stainings. In particular, values obtained with Sirius Red/Fast Green were 3.2- and 2.1-folds higher in the whole colonic wall, and 19.2 and 6.9-folds higher in the *tunica muscularis*, when compared to van Gieson and Sirius Red alone, respectively (Fig 1M and 1N).

### Evaluation of colonic inflammation

**Fig 2:**
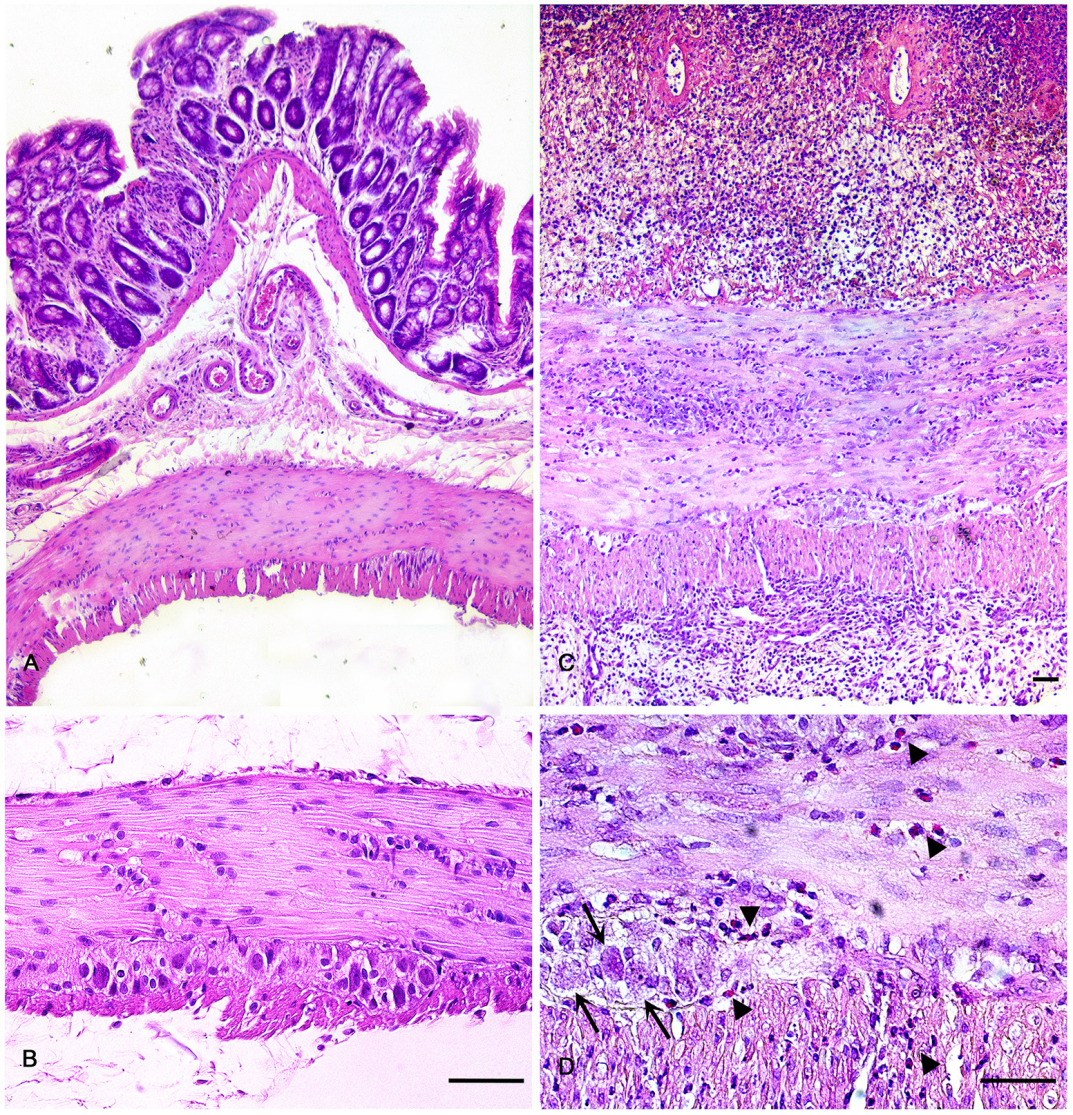
Representative photomicrographs of haematoxylin/eosin-stained full-thickness colonic samples from normal rats (A,B), or rats with DNBS-induced colitis at day 6 (C,D). Normal colon displays a normal morphological architecture of the wall and myenteric ganglia. In the inflamed colon, the following alterations are evident: infiltrated *tunica submucosa*; thickened and infiltrated *tunica muscularis* and *serosa*; vacuolated myenteric ganglia with altered cells and abundant eosinophilic infiltrations along the myenteric ridge (D, arrows and arrowheads, respectively). Scale bars represent 50 μm.

Colonic samples from control rats revealed a normal tissue architecture: the mucosal and submucosal layers were intact and the *tunica muscularis* appeared well conserved and compact, with myenteric ganglia filled of neurons and glial cells. Mucosal and submucosal lesions, consistent with inflammatory alterations, were detected in colonic samples from DNBS-treated rats (Fig 2). In particular, morphologic analysis showed: ulcerated *tunica mucosa-submucosa*, infiltrated and loose *tunica submucosa*, significantly thickened *tunica muscularis* (575±27 μm in the inflamed colon *versus* 171±13 μm in controls, P = 0.0002) and *serosa*. The inflamed colon showed abundant inflammatory infiltrations with high percentage of eosinophils throughout the whole wall (Fig 2).

### Evaluation of collagen fibers in DNBS-inflamed colon

**Fig 3:**
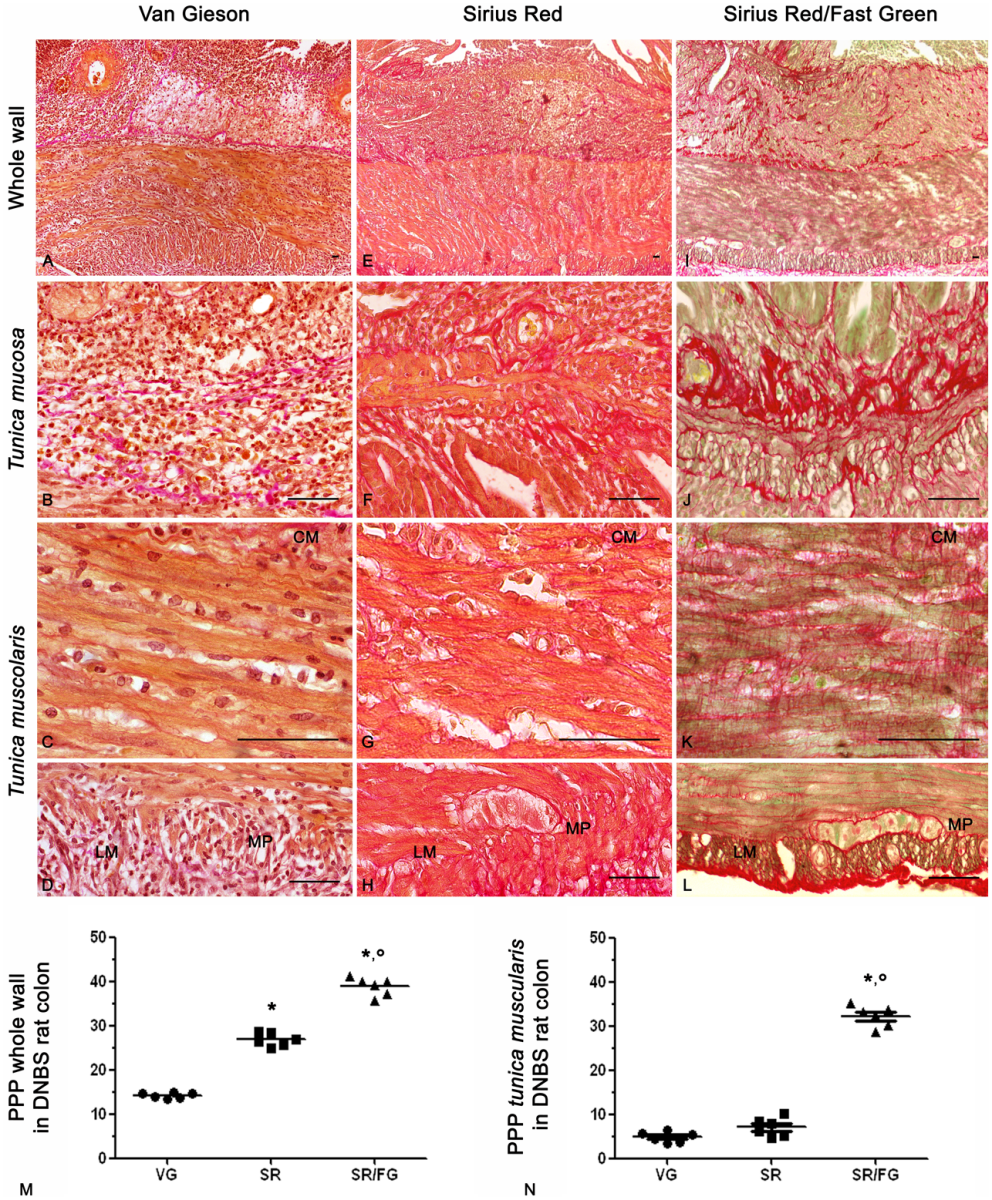
Representative photomicrographs of full-thickness inflamed rat colon showing the distribution pattern of collagen fibers stained with van Gieson (VG, A-D), Sirius Red (SR, E-H) or Sirius Red/Fast Green (SR/FG, I-L). CM and LM: circular and longitudinal muscle, respectively; MP: myenteric plexus. Scale bars represent 50μm. Scatter plots show the percentage of positive pixel (PPP) of collagen ± SEM obtained from 6 rats in the whole colonic wall (M) and *tunica muscularis* (N). *,° P = 0.028 *versus* VG and SR, respectively.

The results obtained with the different histochemical staining protocols in the inflamed colon showed similar sensitivity to that observed in the normal colon (Fig 3). The Sirius Red/Fast Green technique resulted as the most sensitive method to reveal collagen fibers, which appeared well distinguishable from non-collagenic tissues throughout the whole colonic wall (Fig 3I–3L). A consistent collagen deposition was observed in the inflamed colon by this staining at level of the *tunica submucosa* and *muscularis*, mainly along the myenteric ridge and longitudinal muscle layer. In particular, the collagen fiber content, as detected by Sirius Red/Fast Green staining at level of the whole colonic wall, was higher, with a 2.7- and 1.4-fold increment as well as a 6.6- and 4.6-fold increase in the *tunica muscularis*, as compared to van Gieson and Sirius Red, respectively (Fig 3M and 3N). Furthermore, higher amounts of collagen fibers were detected in the inflamed versus normal colon, as indicated by the following increments: 1.74 by van Gieson, 2.20 by Sirius Red, and 1.50 by Sirius Red/Fast Green.

## Discussion

Collagen detection in histological samples represents an important procedure to estimate tissue localization and quantitative expression of connective fibers. This is a very relevant task in pathological conditions such as fibrosis, which results from an imbalance between collagen deposition and reabsorption, generally due to chronic inflammatory processes. In particular, tissue collagen quantification represents an important tool in the clinical diagnosis of fibrosis as well as for outcome prediction and therapy individualization, as in the case of lung [12,26–27], kidney [15,22], liver [28] and intestinal tract [29–30].

Different staining approaches have been proposed to detect and estimate collagen deposition in histological sections, with different degree of satisfaction. Among the histochemical methods, traditional trichrome stains (e.g., Mallory, Masson and van Gieson methods) have been found to underestimate collagen content [7]. Therefore, a picro-sirius stain was set up [5] to implement a more selective method for detecting collagen fibers, which could be appreciated with less fading as compared to van Gieson and subsequently visualized much better by means of polarized light microscopy [6,31–32]. Further studies demonstrated the profitable use of non-polarized light microscopy for evaluating and quantifying Sirius Red-stained collagen in experimental colitis [18] as well as the validation of the Sirius Red-non-polarized strategy for longitudinal clinical studies in chronic nephropathy [15]. The Sirius Red/Fast Green technique then allowed to achieve a better estimation of collagen fibers in formalin-fixed paraffin-embedded sections, due to their red-staining, which stands out from the background of green-stained non-collagen proteins [19].

Based on these considerations, and considering the lack of comparative microscopic evaluations of histochemically stained-collagen, we deemed it interesting to perform a comparative image analysis of three known histochemical techniques for collagen detection (i.e., van Gieson, Sirius Red, Sirius Red/Fast Green) in terms of sensitivity to reveal and quantify collagen deposition in paraffin colonic sections. In order to verify whether the staining properties of the dyes were independent from the histologic appearance of the sample, normal colonic tissues were compared to pathologic specimens obtained from the inflamed colon were examined. In normal colonic samples, the van Gieson technique stained collagen fibers only within the *tunica mucosa* and *submucosa*, while Sirius Red revealed the presence of collagen fibers throughout the wall, highlighting also the thin collagen network within the muscle compartment. The latter observations are in line with the accurate and reliable staining characteristics displayed by Sirius Red in previous studies on the quantification of hepatic collagen [11] or collagen deposition in chronic nephropathy [15]. Under our experimental conditions, the combination of Sirius Red with Fast Green resulted as the best staining procedure to reveal the presence, describe the distribution patterns and perform quantitative estimation of collagen fibers in colonic tissues. Indeed, this double histochemical staining allowed us to selectively label in red more collagen fibers within all layers of the colonic wall, as compared to Sirius Red, as well as to clearly appreciate the thinnest fibers and networks within the *tunica muscularis*, against the brilliant, green background of non-collagen components. Of note, collagen fibers stained by Sirius Red alone appeared as red areas surrounded by a lot of reddish nuances, which could not recorded by the image analysis, being the threshold set up on the red area. By contrast, the double Sirius Red/Fast Green coloration was able to stain the collagen fibers more homogeneously, yielding red areas without blending, and allowed to record overall red-stained collagen areas that were larger than those stained by Sirius Red alone. Besides the best qualitative results, the quantitative estimation of Sirius Red/Fast Green-stained sections by image analysis yielded values that outnumbered significantly those obtained by van Gieson and Sirius Red alone. Of note, the choice of a staining method, which allows to specifically stand out the collagen fibers over the tissue background, plays an important role not only for their morphological identification, but also for a better quantitative assessment as compared to Sirius Red alone. In this respect, the Fast Green dye, which selectively stains non-collagen tissue components, gives rise to a useful color contrast and visualization of red-stained collagen fibers, as well as an optimal threshold for counting the positive pixels and, therefore, to obtain the best quantitative estimation of collagen content by image analysis. Consistently with this view, the Sirius Red/Fast Green staining has been widely used for the *in situ* computer-aided microscopic evaluation of abnormal collagen deposition, such as in kidney [22] and colon [21–23]. Of note, the combination of Sirius Red with Fast Green was introduced since 1985 [19], for collagen staining and quantification in paraffin sections by spectrophotometer analysis of the eluted red dye, and employed in further studies on fibrosis in the liver [33], lung [27] and colon [20,34].

In the present study, a comparative analysis of the collagen-staining capability by the above mentioned techniques was carried out also on the inflamed colon from DNBS-treated rats. The morphological analysis of colitis samples disclosed features compatible with inflammatory lesions with extensive mucosal and submucosal alterations and eosinophil infiltration [30]. In this setting, the inflamed tissues displayed a massive deposition of collagen fibers, and, as in the case of normal colon, the best qualitative and quantitative data were generated by Sirius Red combined with Fast Green. Consistently with previous reports [21,30], our comparative observations confirmed that the Sirius Red/Fast Green combination performs as an optimal histochemical method for collagen detection also in the inflamed colon.

With regard for the detection of collagen in tissue sections, besides the conventional histochemical staining procedures, it must be acknowledged that immunohistochemistry allows a detailed phenotypic analysis of specific connective fibers and their distribution patterns. Nevertheless, multi-antibody immuno-labelling is affected by several disadvantages and pitfalls as compared to histochemistry, such as complex and long-lasting protocols, expensive reagents, and a marked variability in staining patterns of collagen fibers in assays with different commercially available antibodies [35].

In conclusion, the present study provides evidence that histochemical staining carried out by Sirius Red combined with Fast Green represents an excellent method for standing out collagen fibers in paraffin sections of the colon, under both normal and inflammatory conditions, being a technique more sensitive than van Gieson or Sirius Red alone in terms of both morphological and quantitative evaluations.
